# Tuning Electronic Structure and Piezoresistivity of Graphene by Monovacancy Defect Concentration: A First-Principles Investigation

**DOI:** 10.3390/molecules31122007

**Published:** 2026-06-08

**Authors:** Shengsheng Wei, Shuaituan Wang, Ningning Su, Junqiang Wang, Mengwei Li

**Affiliations:** 1 Shanxi Key Laboratory of Graphene Sensing Materials and Devices, North University of China, Taiyuan 030051, China; sswei@nuc.edu.cn (Shengsheng Wei); 20122715@nuc.edu.cn (N.S.); 2 Academy for Advanced Interdisciplinary Research, North University of China, Taiyuan 030051, China; 3 School of Semiconductors and Physics, North University of China, Taiyuan 030051, China; 4 School of Instrument and Electronics, North University of China, Taiyuan 030051, China

**Keywords:** graphene, monovacancy defect, electronic structure, piezoresistive effect

## Abstract

Graphene, with its excellent mechanical and electrical properties, is an ideal candidate material for constructing high-performance piezoresistive sensors. However, lattice defects inevitably introduced during its preparation and transfer processes can significantly alter its electronic structure, thereby affecting the sensing performance of the devices. Based on first-principles calculations, this work systematically investigates the effects of monovacancy defect concentrations ranging from 2% to 8% on the geometric structure, electronic structure, and piezoresistive performance of graphene. The results show that monovacancy defects induce local lattice distortions and bond reconstructions, forming 5–9 non-hexagonal ring structures at defect concentrations of 4% and 8%. In terms of electronic structure, the defects break the lattice symmetry and open a band gap. High concentrations of defects lead to severe overlapping of electronic states, causing the band gap to first increase and then decrease with increasing defect concentration, reaching a maximum value of 0.697 eV at a concentration of 6%. Meanwhile, the defects introduce localized electronic states, enhance the electron localization effect, and render the system p-type doped. Regarding piezoresistive performance, monovacancy defects significantly improve the gauge factor of graphene. At a defect concentration of 6%, the gauge factor reaches 118.23, which is approximately 36 times that of pristine graphene. These findings reveal the microscopic mechanism of strain-dependent electronic modulation in defective graphene and provide theoretical support for defect engineering design in high-performance graphene-based piezoresistive sensors.

## 1. Introduction

Benefiting from advantages such as a simple structure [1,2], low power consumption [3,4], and easy signal readout [5,6], piezoresistive pressure sensors have become core components in fields including aerospace [7], industrial control [8], and medical diagnostics [9]. By leveraging the resistance change in sensitive materials induced by external stress, this type of sensor enables precise capture and electrical conversion of complex mechanical deformations, thereby occupying an important position in high-performance sensing technology. Among numerous candidate sensitive materials, graphene is regarded as an ideal sensing material for fabricating next-generation piezoresistive pressure sensors due to its excellent mechanical strength [10], extremely high carrier mobility [11], and good flexibility [12]. Therefore, extensive research efforts have been devoted to optimizing the synthesis and processing strategies of graphene to fully harness its application potential [13,14]. However, the zero-bandgap electronic structure of graphene limits its application in semiconductor sensors; moreover, lattice defects are inevitably introduced during its preparation and transfer processes [15,16], which also affect its electrical properties.

In recent years, defect engineering, as a powerful regulatory approach, has introduced specific types of defects into the lattice through methods such as high-energy particle/electron beam irradiation [17,18], plasma treatment [19], and chemical etching [20], thereby breaking the periodic symmetry of graphene and effectively modulating its band structure and electrical transport properties. To break the constraints of graphene’s intrinsic properties, researchers have developed a variety of effective strategies to modulate its piezoresistive properties. For example, Lim et al. utilized CVD-grown multilayer graphene thermally transferred onto a silicon wafer to fabricate a thin-film piezoresistive pressure sensor, achieving a gauge factor of up to 17.5 [21]. Hafiz et al. obtained graphene with different defect densities by tuning the growth temperature of HFTCVD graphene, fabricated flexible pressure sensors, and found that the higher the defect density, the higher the piezoresistive sensitivity, reaching 0.0045 kPa^−1^ at 750 °C [22]. Haniff et al. used NH_3_/Ar plasma treatment to nitrogen-dope graphene and fabricated a high-performance flexible pressure sensor, achieving a gauge factor that is approximately one order of magnitude higher than that of the untreated sample [23]. Lv et al. alternately coated graphene oxide and polypyrrole onto a polyurethane sponge skeleton via layer-by-layer self-assembly, utilizing the changes in contact area and conductive pathways of the fragmented conductive layer upon compression to fabricate a piezoresistive pressure sensor with a sensitivity as high as 0.79 kPa^−1^ [24]. Although extensive research has been conducted on the modulation of the piezoresistive properties of graphene, there is still a lack of systematic study on the most common monovacancy defect in graphene [25], and the underlying micro-mechanism linking defect concentration, structural reconstruction, electronic evolution, and piezoresistive response is still unclear.

In this study, we constructed defective graphene models with different monovacancy concentrations and employed first-principles calculations based on density functional theory to investigate the effects of varying monovacancy concentrations on the electronic and geometric structures of graphene. We quantified the influence of monovacancy concentration on the piezoresistive effect of graphene. This research provides a theoretical basis for understanding the mechano-electrical coupling behavior of graphene under defect modulation and lays the foundation for the design of high-performance piezoresistive sensors based on defect engineering.

## 2. Results and Discussion

### 2.1. Structural Properties

**Figure 1:**
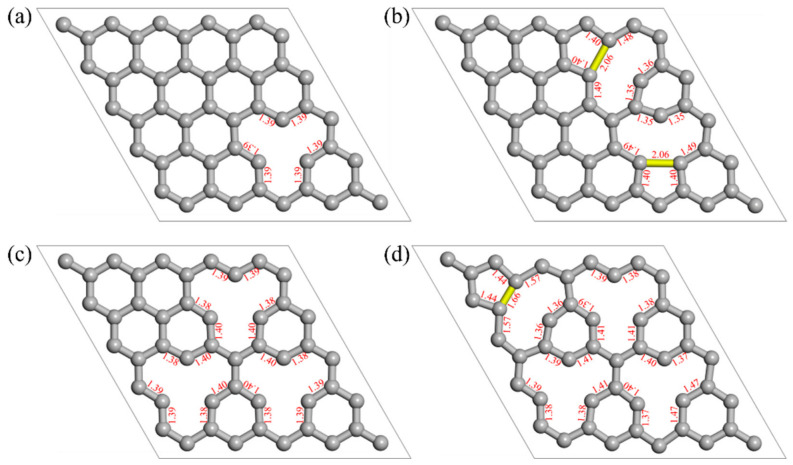
Geometrically relaxed structures of defective graphene with monovacancy concentrations of (**a**) 2%, (**b**) 4%, (**c**) 6%, and (**d**) 8%. The yellow bonds represent the newly formed chemical bonds resulting from structural optimization.

To reveal the regulation mechanism of monovacancy defects on the properties of graphene, the geometric structures of graphene with different defect concentrations were first analyzed. As shown in Figure 1, at a defect concentration of 2%, the introduction of a monovacancy primarily induces a slight lattice distortion in the local region. The C–C bond lengths around the defect change from 1.42 Å to 1.39 Å, with a relatively small overall variation and uniform distribution, indicating that the structural perturbation is mainly confined to the vicinity of the defect and has a limited impact on the overall lattice. As the defect concentration increases, the structural distortion effect gradually intensifies, and the lattice symmetry is further disrupted. The defect-induced structural perturbation is no longer confined to the vicinity of a single vacancy but extends outward, manifested as a marked increase in the inhomogeneity of bond length distribution and a greater deviation of local atoms from their equilibrium positions. Notably, at defect concentrations of 4% and 8%, the bond length in the yellow region shown in the figure decreases from the initial 2.46 Å to 2.06 Å and 1.66 Å, respectively, indicating that bond reconstruction may have occurred in this region, forming a 5–9 non-hexagonal ring configuration, which is also one of the more common defect models [26,27]. This reconstruction process can be attributed to the degeneracy lifting of local electronic states induced by unsaturated carbon atoms near the defect, thereby triggering a Jahn–Teller distortion [28,29], which causes the system to reduce symmetry and reform bonds to lower the total energy, ultimately forming a more stable local structure. This structural reconstruction and distortion induced by monovacancy defects not only alter the local atomic arrangement of graphene but also significantly impact its electronic state distribution and carrier transport pathways.

On the basis of clarifying the influence of monovacancy defects on the geometric structure of graphene, we further introduce the defect formation energy from an energy perspective to characterize the ease of defect formation and the thermodynamic stability of the system. Its calculation Equation is as follows [30]:

(1) $$E_{f}=E_{defect}-E_{pristine}+n\times \mu_{c}$$

where *E_defect_* represents the total energy of the system containing defects, *E_pristine_* represents the total energy of the perfect crystal, *n* represents the number of C atoms added or removed relative to the perfect crystal, and *μ_C_* represents the chemical potential of a C atom.

The calculated formation energies for different monovacancy concentrations are as follows: As the vacancy concentration increases, the total formation energy of the system gradually increases: at concentrations of 2%, 4%, 6%, and 8%, the total formation energies are 8.0, 15.0, 23.8, and 29.4 eV, respectively, indicating that the overall thermodynamic stability of the system gradually decreases at high defect concentrations, and introducing more vacancies requires overcoming a higher energy barrier. However, in terms of the average formation energy per vacancy, the values at concentrations of 2%, 4%, 6%, and 8% are 8.0, 7.5, 7.9, and 7.35 eV, respectively. It can be seen that the average formation energies at 4% and 8% concentrations are significantly lower than those at 2% and 6% concentrations. This is closely related to the essential differences in the local defect structures of the two groups. At concentrations of 4% and 8%, the unsaturated carbon atoms around the defects induce the formation of new chemical bonds between adjacent atoms, and the energy released by bond reconstruction effectively lowers the total energy of the system, resulting in relatively lower average formation energy per vacancy for these two configurations.

### 2.2. Electrical Properties of Intrinsic Graphene

**Figure 2:**
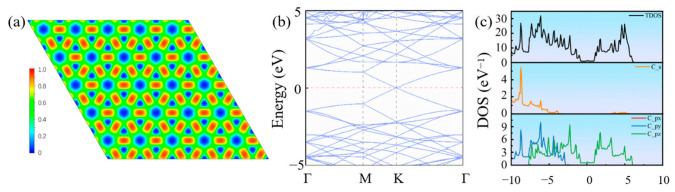
Electrical properties of intrinsic graphene: (**a**) local charge density, (**b**) band structure, (**c**) density of states. The C_px and C_py curves in (**c**) overlap, so the red line is not visible.

Before analyzing the influence of different monovacancy concentrations on the electrical properties of graphene, it is necessary to first calculate the electrical properties of intrinsic graphene. Figure 2 presents the calculated local charge density, band structure, and density of states of intrinsic graphene. The local charge density map shows that charge is mainly concentrated in the C-C bond regions, with a uniform overall distribution, reflecting the strong σ covalent bond characteristics formed by sp^2^ hybridization and the complete lattice periodicity [31]. The band structure diagram reveals that the valence band and conduction band of intrinsic graphene cross linearly at the K point of the Brillouin zone, with the crossing point located at the Fermi level and a zero bandgap [32], exhibiting typical Dirac cone linear dispersion characteristics, where carriers behave as massless Dirac fermions. Density of states analysis further indicates that the total density of states near the Fermi level approaches zero, corresponding to the linear dispersion relation of the Dirac cone. The electronic states near the Fermi level are primarily contributed by pz orbitals, which are the core source of the delocalized π bonds, while the contributions from px and py orbitals are mainly distributed in the lower energy regions, collectively forming the in-plane σ bond skeleton. The above results are in good agreement with the electronic structure characteristics of intrinsic graphene reported in the literature and can serve as a reliable reference for comparative analysis of subsequent defective systems.

### 2.3. Electrical Properties of Defective Graphene with Different Monovacancy Concentrations

**Figure 3:**
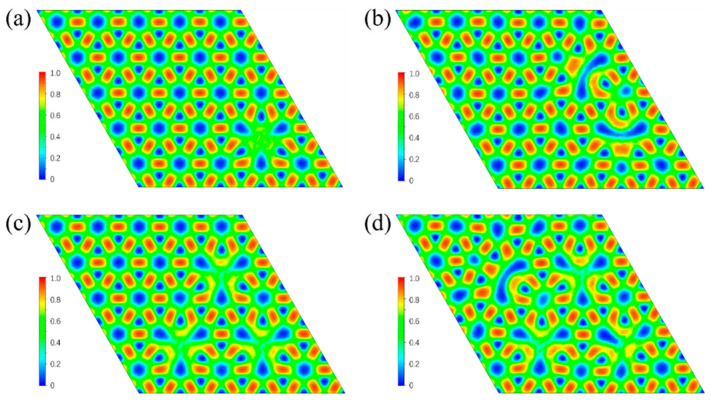
Local charge density distributions of graphene with (**a**) 2%, (**b**) 4%, (**c**) 6%, and (**d**) 8% monovacancy concentrations.

To investigate the redistribution characteristics of electrons in the lattice after defect introduction, we calculated the local charge density of defective graphene with monovacancies at different concentrations, as shown in Figure 3. At a monovacancy concentration of 2%, the electron charge decreases around the missing C atom, forming local electron holes and exhibiting a slight electron localization effect, while most regions of the lattice still maintain a uniform charge distribution. As the monovacancy concentration increases, the inhomogeneity of the local charge density gradually intensifies, the electron localization effect in the defect regions becomes more pronounced, and the interaction between defects expands the range of electron redistribution, progressively disrupting the overall uniformity of the lattice. At concentrations of 4% and 8%, significant charge accumulation can be observed at positions A, B, and D, respectively, for each concentration. Combined with structural analysis, covalent bonds may have formed at these positions, further stabilizing the electronic structure of the defect regions. Overall, as the monovacancy concentration increases, the localization effect and electron redistribution trend are significantly enhanced, providing a microscopic basis for understanding the regulation mechanism of defects on the electrical properties of graphene.

**Figure 4:**
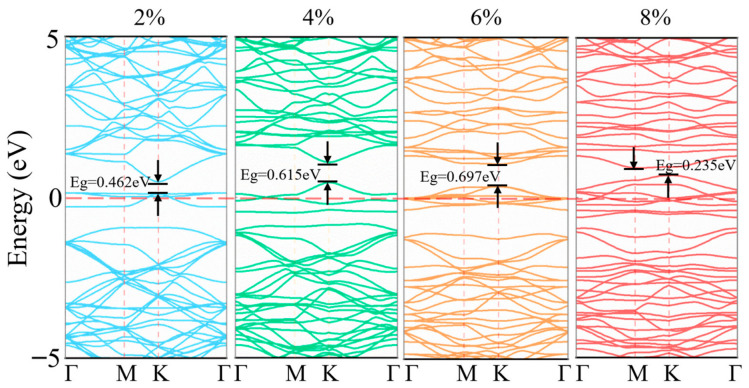
Band structures of defective graphene with different monovacancy concentrations. The upward and downward arrows represent the conduction band minimum and the valence band maximum, respectively, with the band gap in between.

As shown in Figure 4, we calculated the band structures of defective graphene with different concentrations of monovacancy defects. The introduction of monovacancies breaks the equivalent symmetry between lattice sites, eliminates the band degeneracy at the Dirac point, and opens a band gap near the Fermi level. As the vacancy concentration increases, the band gap first increases and then decreases, reaching a maximum value of 0.697 eV at a concentration of 6%. At a monovacancy defect concentration of 8%, strong coupling between defects leads to severe overlapping of localized electronic states, which in turn causes energy level broadening and convergence of band edges, ultimately reducing the band gap. Meanwhile, the localized flat bands near the Fermi level become increasingly pronounced—such flat bands originate from the localized electronic states corresponding to dangling bonds of unsaturated atoms around the vacancies, exhibiting weak band dispersion. The higher the defect concentration, the greater the number and density of localized states, reflecting a continuously enhanced electron localization effect. Defects disrupt the periodic potential field of graphene, increasing the effective mass of carriers and significantly reducing their mobility, consistent with the degradation of electrical transport properties in defective systems. When the defect concentration reaches 8%, the conduction band minimum and valence band maximum are no longer located at the same k-point in the Brillouin zone, and the system transitions from a direct band gap to an indirect band gap, indicating that the interaction between defects is strong enough to alter the momentum-space distribution of band edges. Furthermore, the Fermi level continuously shifts downward below the valence band maximum, suggesting that monovacancy defects introduce effective hole doping into the system, gradually endowing the material with p-type semiconductor characteristics. In summary, monovacancy defects achieve multi-dimensional regulation of the band structure of graphene by introducing localized states, breaking lattice symmetry, and enhancing defect-defect coupling. These changes lay an important electronic structure foundation for the subsequent modulation of piezoresistive effects.

**Figure 5:**
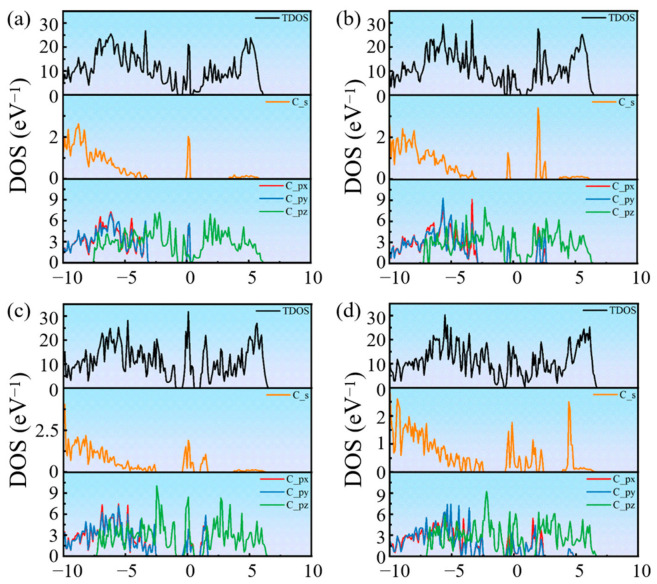
Total density of states and partial density of states of defective graphene with (**a**) 2%, (**b**) 4%, (**c**) 6%, and (**d**) 8% monovacancy concentrations.

To further analyze the influence of monovacancy defects on the electrical properties of graphene from the perspective of electronic state distribution, the total density of states (DOS) and partial density of states (PDOS) at different defect concentrations were calculated, and the results are shown in Figure 5. The total DOS results reveal that as the monovacancy concentration increases, pronounced sharp peaks gradually appear near the Fermi level, which is one of the most distinctive features of defective systems. This observation indicates that monovacancies introduce a large number of localized electronic states into the system, primarily originating from the dangling bonds of unsaturated carbon atoms around the vacancies, and corresponds to the flat-band characteristics in the band structure, reflecting the continuous enhancement of electron localization. Furthermore, at higher defect concentrations, the overall distribution range of the DOS peaks broadens significantly, and the asymmetry between the two sides of the Fermi level increases, indicating a higher degree of discretization of electronic states induced by defects and further disruption of system symmetry. This evolution of the electronic structure, characterized by the combined effect of enhanced localization and level broadening, suggests that monovacancy defects not only alter the distribution pattern of electronic states but also significantly impact carrier transport behavior. The PDOS results show that as the vacancy concentration increases, sharper peaks gradually appear in the PDOS of the s-orbital near the Fermi level, while the original symmetry and consistency of the px and py orbitals are markedly disrupted. This phenomenon indicates that the local structural distortion induced by defects breaks the ideal sp^2^ hybridization state, causing the s-orbitals, which originally primarily participated in σ-bond formation, to begin contributing to the electronic states near the Fermi level. This orbital rehybridization behavior reflects a significant reconstruction of the electronic structure in the defect regions and further enhances the electron localization characteristics, thereby exerting a profound influence on the electrical properties of the system.

### 2.4. Electronic Band Structure of Graphene with Various Monovacancy Concentrations Under Strain

In graphene-based piezoresistive sensors, external forces are transferred to the graphene sensing region through the device structure, causing deformation and strain in the graphene layer. The applied strain changes the C-C bond lengths and the interaction between orbitals, which further affects the electronic transport behavior and electrical properties of graphene. Therefore, studying the evolution of the electronic structure under strain is important for understanding the sensing mechanism of graphene-based devices.

**Figure 6:**
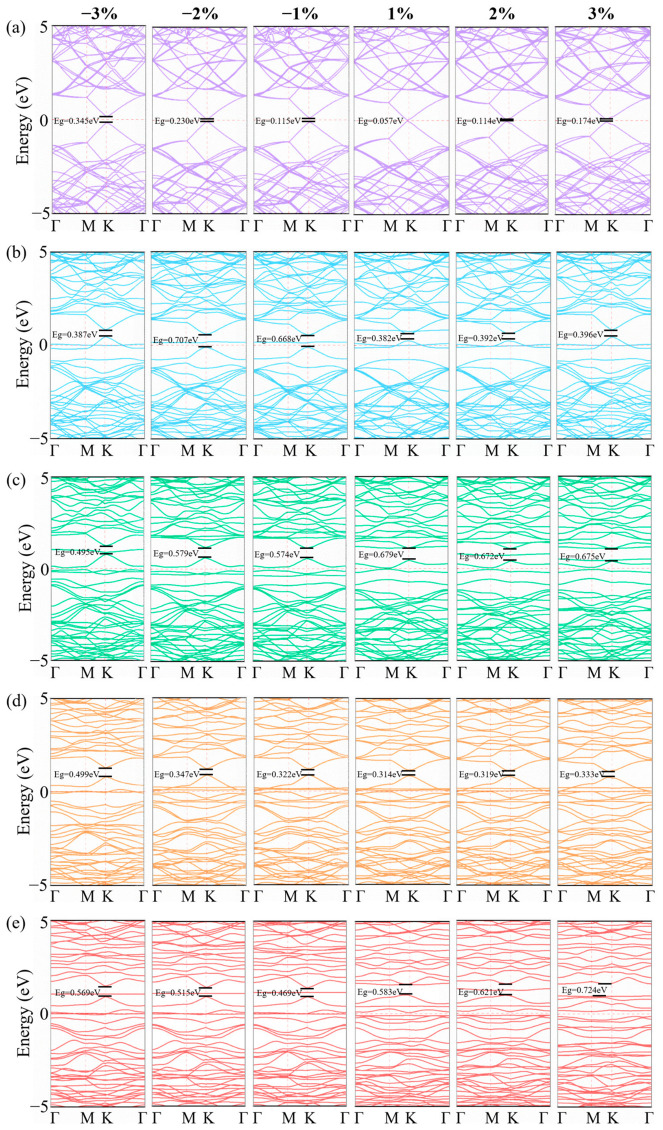
Band structures of graphene with different monovacancy concentrations under strains ranging from −3% to 3%. (**a**–**e**) correspond to the band structure of graphene with monovacancy concentrations of 0%, 2%, 4%, 6% and 8%, respectively.

**Figure 7:**
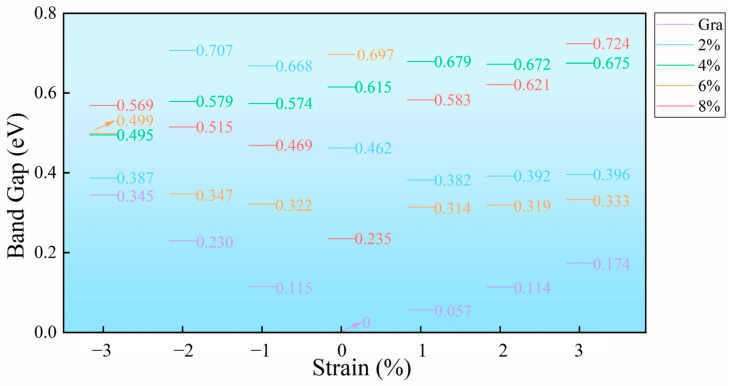
Bandgap of graphene with different monovacancy concentrations under −3% to 3% strain.

In this work, uniaxial strains ranging from −3% to 3% were applied to graphene systems with different monovacancy concentrations to simulate the strain conditions that may occur during device operation. The corresponding band structures are shown in Figure 6, and the bandgap variations under different strain conditions are summarized in Figure 7.

As shown in Figure 6a, the electronic band structure of intrinsic graphene remains relatively stable under both compressive and tensile strains, and the Dirac cone near the Fermi level is well preserved throughout the entire strain range. Although the linear dispersion characteristic is maintained, the bandgap gradually increases with increasing strain magnitude under both tensile and compressive conditions (Figure 7), with compressive strain showing a more obvious enhancement effect. This behavior is mainly related to strain-induced variations in C-C bond lengths and π-π* orbital coupling, which slightly modify the electronic states around the Dirac point while preserving the overall electronic characteristics of graphene.

For graphene with a 2% monovacancy concentration, the band structure near the Fermi level changes significantly compared with intrinsic graphene (Figure 6b). Two defect-induced localized states appear around the Fermi level, indicating that the vacancies disrupt the local sp^2^ bonding network and introduce dangling-bond states. Under compressive strain, the reduced lattice spacing strengthens the interaction between defect wavefunctions, causing the defect levels to gradually approach and partially merge. As shown in Figure 7, the bandgap increases from 0 to −2% strain but decreases obviously at −3% strain and becomes smaller than that of the unstrained system. This phenomenon may result from excessive lattice compression, which enhances defect-state overlap and promotes the formation of mid-gap states. Under tensile strain, the coupling between defect states weakens, leading to the splitting of the defect levels and their gradual movement toward the valence and conduction bands. Although the bandgap still increases with increasing tensile strain, all strained systems exhibit smaller bandgaps than the unstrained graphene, suggesting that the localized defect states partially suppress the strain-induced bandgap opening effect.

When the vacancy concentration increases to 4%, more obvious lattice reconstruction occurs around the defect regions, leading to stronger perturbation of the original hexagonal symmetry. Compared with the 2% vacancy system, the band dispersion near the Fermi level becomes flatter (Figure 6c), indicating enhanced localization of electronic states, which is also consistent with the local charge density distribution shown in Figure 4. Under tensile strain, the bandgap shows only a slight increase and remains nearly constant at about 0.675 eV (Figure 7), indicating that the defect-localized states dominate the electronic structure near the Fermi level and weaken the strain modulation effect. Under compressive strain, the bandgap decreases continuously and reaches the minimum value at −3% strain. In addition, obvious defect-related mid-gap states appear under both tensile strain and large compressive strain (−3%), indicating that strain further modifies the coupling and hybridization of localized defect orbitals.

For graphene with a 6% vacancy concentration, the Dirac-cone-like feature is still partially preserved but becomes noticeably weaker than that of the lower vacancy concentration systems (Figure 6d), indicating that the long-range π-conjugation network is increasingly disrupted by vacancy-induced disorder. Under both tensile and compressive strains, the bandgap gradually increases with increasing strain magnitude (Figure 7). However, all strained systems still show smaller bandgaps than the unstrained structure, suggesting that defect-localized states continue to dominate the electronic structure near the Fermi level. Under tensile strain, the increased lattice spacing weakens orbital interactions between carbon atoms and further breaks the lattice symmetry, causing the valence band maximum (VBM) and conduction band minimum (CBM) to shift toward the K-M direction in reciprocal space. Under compressive strain, enhanced orbital overlap and lattice distortion also contribute to the increase in bandgap.

At the highest vacancy concentration of 8%, the band structure becomes highly disordered and dominated by defect-derived electronic states (Figure 6e). The sp^2^ conjugated network is severely disrupted, and a large number of nearly flat bands appear around the Fermi level, indicating strong localization of electronic states. The distinction between the valence and conduction bands becomes less clear, while multiple defect-related mid-gap states emerge under strain modulation. Under tensile strain, the redistribution of electronic states becomes more obvious due to enhanced lattice symmetry breaking and bond stretching. In particular, both the VBM and CBM shift evidently toward the midpoint of the M-K high-symmetry path. Meanwhile, compressive strain enhances the overlap and hybridization of localized defect orbitals. Compared with the lower vacancy concentration systems, the electronic structure at 8% vacancy concentration is dominated more strongly by defect-state interactions than by the intrinsic Dirac electronic characteristics of graphene.

### 2.5. Piezoresistive Effect

The piezoresistive effect is an important parameter for evaluating piezoresistive pressure sensors and is usually characterized by the gauge factor (GF), which reflects the sensitivity of resistance change under uniaxial strain.

**Figure 8:**
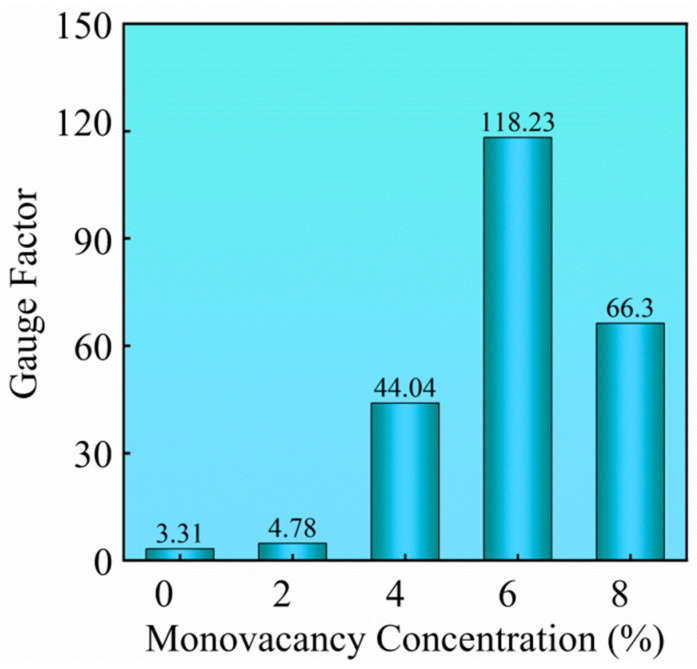
Gauge factor of intrinsic graphene and graphene with different monovacancy concentrations.

Yokaribas et al. have shown that the presence of defects can indeed enhance the piezoresistive effect of graphene [33]. To quantify the influence of different monovacancy concentrations (2%, 4%, 6%, 8%), we calculated the corresponding piezoresistive effect, and the results are shown in Figure 8. Overall, monovacancy defects can significantly improve the piezoresistive response of graphene. The GF increases from 3.31 for intrinsic graphene to 118.23 at a vacancy concentration of 6%, which is about 36 times higher than that of pristine graphene. Combined with the strain-dependent band structure analysis, this enhancement is mainly related to the increased sensitivity of the electronic structure after vacancy defects are introduced. For intrinsic graphene, the Dirac cone remains relatively stable under strain, resulting in only limited changes in electronic transport properties and a relatively low GF. After introducing monovacancy defects, localized states and lattice distortion appear near the Fermi level, making the electronic structure more sensitive to strain. At moderate defect concentrations (4–6%), strain induces obvious defect-level splitting, band-edge shifts, and orbital coupling changes, which further strengthen the modulation of the Fermi velocity and improve the piezoresistive response.

However, the GF does not continuously increase with defect concentration. When the vacancy concentration reaches 8%, the GF decreases to 66.3. This decrease is mainly caused by the excessive overlap and localization of defect states at high defect concentrations. The strongly distorted sp^2^ conjugated network weakens the intrinsic Dirac electronic characteristics of graphene, while excessive localized states reduce the modulation effect of strain on the electronic structure. Therefore, an appropriate monovacancy concentration is more beneficial for improving the piezoresistive performance of graphene. These results suggest that defect engineering has good potential for tuning the sensing performance of graphene and may provide useful guidance for graphene-based piezoresistive pressure sensors in flexible and wearable electronic devices.

## 3. Computational Methods

In this study, a 5 × 5 graphene supercell containing 50 carbon atoms with a lattice constant of 12.30 Å was adopted as the computational model. To avoid the interference of interlayer interactions on the electronic structure under periodic boundary conditions, a vacuum layer of 15 Å was set along the direction perpendicular to the graphene plane. First-principles calculations based on density functional theory(DFT) [34,35] were performed using the Vienna Ab Initio Simulation Package (VASP) [36], in which the electron-ion interactions are described by the projector augmented wave (PAW) method [37]. The exchange–correlation functional was treated using the generalized gradient approximation (GGA) with the Perdew–Burke–Ernzerhof (PBE) functional [38]. The plane-wave cutoff energy was set to 400 eV. A 9 × 9 × 1 k-point mesh generated using the Gamma-centered Monkhorst–Pack method was used to sample the Brillouin zone, and this setup accurately describes the band structure and density of states of the system (convergence tests were performed by employing a denser 11 × 11 × 1 k-point mesh. In both cases, the total energy variation was less than 1 meV/atom, demonstrating that the selected computational parameters provide sufficient accuracy for the present calculations). During geometry optimization, the energy convergence criterion was set to 1 × 10^−5^ eV, and the maximum atomic force convergence criterion was set to 0.01 eV/Å. The above parameters have been tested for convergence, ensuring the reliability of subsequent analyses of the electronic structure and piezoresistive effect.

**Figure 9:**
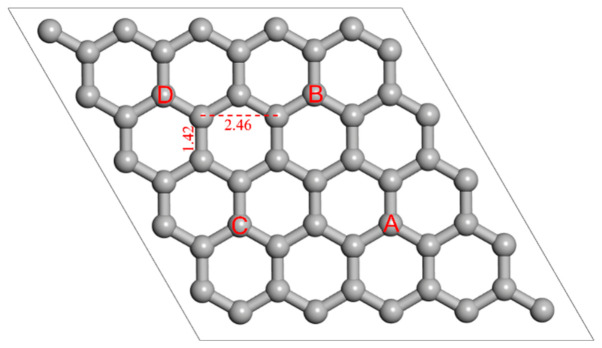
Schematic diagram of the construction of defective graphene models with different monovacancy concentrations. Defective graphene systems with monovacancy concentrations of 2%, 4%, 6%, and 8% are constructed by sequentially removing carbon atoms at sites A, B, C, and D, respectively.

To investigate the effects of different monovacancy concentrations, we constructed defective graphene based on the pristine graphene structure by removing carbon atoms one by one. The specific model construction is shown in Figure 9. By sequentially removing carbon atoms at positions A, B, C, and D, graphene models with monovacancy concentrations of 2%, 4%, 6%, and 8% were obtained, respectively, for subsequent studies.

The vacancy configurations considered in this study are constructed based on periodic supercell models, wherein the vacancies are arranged in a regular and symmetric distribution. The initial purpose of this method is to establish a systematic and controllable framework, mainly for exploring the influence of vacancy concentration on the electronic structure and piezoresistive response of graphene; in addition, there are precedents for this method in theoretical literature related to defect engineering of two-dimensional materials [39,40]. Nevertheless, this periodic vacancy superlattice model is not without its limitations; for instance, it is incapable of capturing the changes in graphene properties that arise from the interactions between adjacent vacancies.

The core working principle of piezoresistive pressure sensors is the piezoresistive effect. The piezoresistive effect refers to the change in electrical resistance of a material when subjected to external mechanical stress, and this change primarily originates from alterations in the material’s geometric dimensions and its internal resistivity. Its defining Equation is [41]:

(2) $$GF=\frac{\Delta R/R}{\varepsilon }$$

According to [42]:

(3) $$R=\rho \cdot \frac{L}{S}$$

In the Equation, *R* represents resistance, *ρ* represents resistivity, *L* represents the length of the conductor, and *S* represents the cross-sectional area. Taking the logarithm of both sides of the Equation and then performing total differentiation yields:

(4) $$\frac{\Delta R}{R}=\frac{\Delta L}{L}-\frac{\Delta S}{S}+\frac{\Delta \rho }{\rho }$$

For the case of applying a uniaxial strain along the in-plane horizontal direction, the thickness *h* of monolayer graphene remains essentially unchanged, thus yielding the following expression.

(5) $$\frac{\Delta S}{S}=\frac{h\cdot \Delta W}{h\cdot w}=\frac{\Delta W}{w}$$

In the Equation, *w* represents the cross-sectional width of graphene. Given that resistivity is inversely proportional to the square of the Fermi velocity, the following expression is obtained [43]:

(6) $$\rho =\frac{1}{n_{e}\cdot e\cdot \mu_{e}}=\frac{c}{v_{F}^{2}}$$

In the above Equation, the symbol $n_{e}$ represents the carrier concentration, $\mu_{e}$ represents the carrier mobility, e denotes the electron charge, and *c* is a constant term.

If the applied strain is small and Δ*V_f_* is much smaller than *V_f_*, the following expression can be derived:

(7) $$\frac{\Delta \rho }{\rho }=-2\frac{\Delta v_{f}}{v_{f}}$$

By substituting Equations (5) and (7) into Equation (4), we obtain the following result:

(8) $$\frac{\Delta R}{R}=\frac{\Delta l}{l}-\frac{\Delta w}{w}-2\frac{\Delta v_{f}}{v_{f}}$$

Based on the fundamental definitions of strain and Poisson’s ratio, the following relationship can be obtained:

(9) $$\varepsilon =\frac{\Delta l}{l}$$

(10) $$\frac{\Delta w}{w}=-\nu \frac{\Delta l}{l}$$

where *ε* and *ν* represent strain and Poisson’s ratio, respectively, then Equation (8) can be expressed as:

(11) $$\frac{\Delta R}{R}=\varepsilon +\nu \varepsilon -2\frac{\Delta v_{f}}{v_{f}}$$

Substituting Equation (11) into Equation (2), we finally obtain [44]:

(12) $$GF=1+\nu -2\frac{\Delta v_{f}/v_{f}}{\varepsilon }$$

According to Equation (12), the GF is closely related to the strain-induced variation in the Fermi velocity. The Fermi velocity can be calculated by [45]:

(13) $$V_{f}=\frac{1}{\hbar }\frac{dE}{dK}$$

By substituting Equation (13) into Equation (12), the GF can be expressed as:

(14) $$GF=1+\upsilon -2\frac{{\Delta E}_{1}-\Delta E}{\Delta E\times \varepsilon }$$

Here, ${\Delta E}_{1}$ and ΔE are the energy differences near the Dirac point before and after strain application, respectively, which can be obtained from the band structures.

In summary, the defective graphene models with different monovacancy concentrations constructed based on first-principles calculations, combined with the theoretical derivation of the piezoresistive effect, provide theoretical support for subsequent research on the piezoresistive properties of defective graphene.

## 4. Conclusions

Based on first-principles calculations, this study systematically investigates the effects of monovacancy defect concentration on the geometric structure, electronic properties, and piezoresistive behavior of graphene. The results show that monovacancy defects induce local lattice distortion and bond reconstruction, forming 5–9 non-hexagonal ring structures at defect concentrations of 4% and 8%, while the formation energy gradually increases with increasing defect concentration. In terms of electronic structure, vacancy defects break the lattice symmetry of graphene. As the defect concentration increases, the enhanced overlap and localization of defect states cause the bandgap to first increase and then decrease, reaching a maximum value of 0.697 eV at a defect concentration of 6%. Meanwhile, the Fermi level gradually shifts downward, indicating enhanced p-type doping behavior. Under uniaxial strain ranging from −3% to 3%, intrinsic graphene maintains a relatively stable Dirac-cone structure, whereas defective graphene exhibits more complex band structure evolution due to defect-state localization, orbital hybridization, and lattice distortion. In particular, strain can induce defect-level splitting, mid-gap-state formation, and shifts in the VBM and CBM toward the K-M direction in reciprocal space. More importantly, monovacancy defects significantly enhance the piezoresistive response of graphene, with the maximum gauge factor reaching 118.23 at a defect concentration of 6%, which is approximately 36 times higher than that of pristine graphene. These findings provide theoretical support for understanding the strain-dependent electronic modulation mechanism of defective graphene and for the design of high-performance graphene-based piezoresistive sensors.

## Data Availability

The data that support the findings of this study are available from the corresponding author upon reasonable request.
